# Fin whale song variability in southern California and the Gulf of California

**DOI:** 10.1038/s41598-017-09979-4

**Published:** 2017-08-31

**Authors:** Ana Širović, Erin M. Oleson, Jasmine Buccowich, Ally Rice, Alexandra R. Bayless

**Affiliations:** 1 Scripps Institution of Oceanography, University of California San Diego, La Jolla, CA 92093-0205 USA; 2 Pacific Islands Fisheries Science Center, NOAA Fisheries, Honolulu, HI 96818 USA; 3 Joint Institute for Marine and Atmospheric Research, University of Hawaii at Manoa, Honolulu, HI 96822 USA

## Abstract

Songs are distinct, patterned sounds produced by a variety of animals including baleen whales. Fin whale songs, which consist of short pulses repeated at regular interpulse intervals (IPIs), have been suggested as a tool to distinguish populations. Fin whale songs were analyzed from data collected from 2000–2012 in Southern California and from 2004–2010 in the Gulf of California using autonomous acoustic recorders. IPIs were measured for each identifiable song sequence during two random days of each month with recordings. Four distinct song types were identified: long doublet, short doublet, long triplet, and short triplet. Long and short doublets were the dominant songs in Southern California, while long and short triplets were dominant in the Gulf of California. An abrupt change in song type occurred in both areas during the monitoring period. We argue that each song type is unique to a population and these changes represent a shift in the primary population in the monitoring area. Occasional temporal and spatial song overlap indicated some exchange or visitation among populations. Fin whales appear to synchronize and gradually modify song rhythm over long time scales. A better understanding of the evolutionary and ecological importance of songs to fin whale populations is needed.

## Introduction

Songs are distinctive sounds or sequences of sounds produced in a repeated pattern^1^. They can also be defined as stereotyped signals that serve a reproductive function^2^. Songs have been documented in a variety of animals, including insects, birds, and mammals. Songs can be innate, whereby they do not need to be heard and learned through imitation, but when dialects exist they are typically acquired via vocal learning, which is the modification of sounds through experience with other individuals^3, 4^. The function of song has been most extensively studied in oscine birds, which use songs for mate attraction or intrasexual competition^2^. As a reproductive display, therefore, song is primarily shaped by sexual selection^5^.

Songs are used by a number of baleen whale species^1, 6–8^. Complexity of baleen whale songs ranges from highly variable songs of humpback whales (*Megaptera novaeangliae*)^6^, to less complex blue whale (*Balaenotpera musculus*) songs^1^. Fin whale (*B*. *physalus*) song consists of low frequency, 20 Hz pulses arranged in a regular sequence^9, 10^. These songs are produced by males and are postulated to have a reproductive function^7^. In addition, there is evidence of regional specificity in songs that may be linked to population structure. The specificity can show itself in the presence of higher frequency pulse components^11, 12^, or in the interpulse interval (IPI) durations of a song sequence, with all members in an area seemingly matching the same frequency and IPI features^9, 10, 13^.

Based on their IPIs, fin whale songs can be classified into three broad categories: singlets, doublets, and triplets^9, 10, 14^. Singlets are songs with only one distinct IPI. Doublet songs have two alternating IPIs of different lengths. Triplets also have at least two distinct IPIs, where one of them may be repeated two or more times.

The distribution and population structure of fin whales appears to be more complex than previously thought. Their migration patterns do not conform to the generally posited baleen whale seasonal migration from summer feeding grounds to winter breeding grounds as they have more varied movements^15^. Additionally, in the Northeastern Pacific there is a resident population of fin whales in the Gulf of California^16^, and possibly another in Southern California^17^. Until recently, a single northern hemisphere fin whale subspecies was recognized, but mitogenomic evidence now points to two separate subspecies, one in the North Pacific and one in the North Atlantic^18^. It is unclear whether there will be any further subdivision to populations in the North Pacific, but song has been suggested as a possible tool for undertaking such delineation.

Previous analyses of fin whale acoustic records have generally focused on broad spatial and temporal patterns of call occurrence, or more detailed call descriptions, while grouping all 20 Hz calls into a single category^11, 17, 19–24^. Based on such analyses, we know fin whales produce 20 Hz calls year-round in Southern California, with a decrease in calling during the summer^17^. In contrast, these calls peak in the Gulf of California in the summer and fall^25^. In addition to patterned songs, fin whales also use 20 Hz pulses in irregular and call-counter call patterns that likely have a different social context than song^26^ and do not show obvious differences across populations. Thus analysis of simple presence of 20 Hz signals, even though useful for indication of fin whale presence, may not provide information on population structure or ecology of the species. A more detailed analysis of calling patterns is needed to reveal those elements.

**Figure 1:**
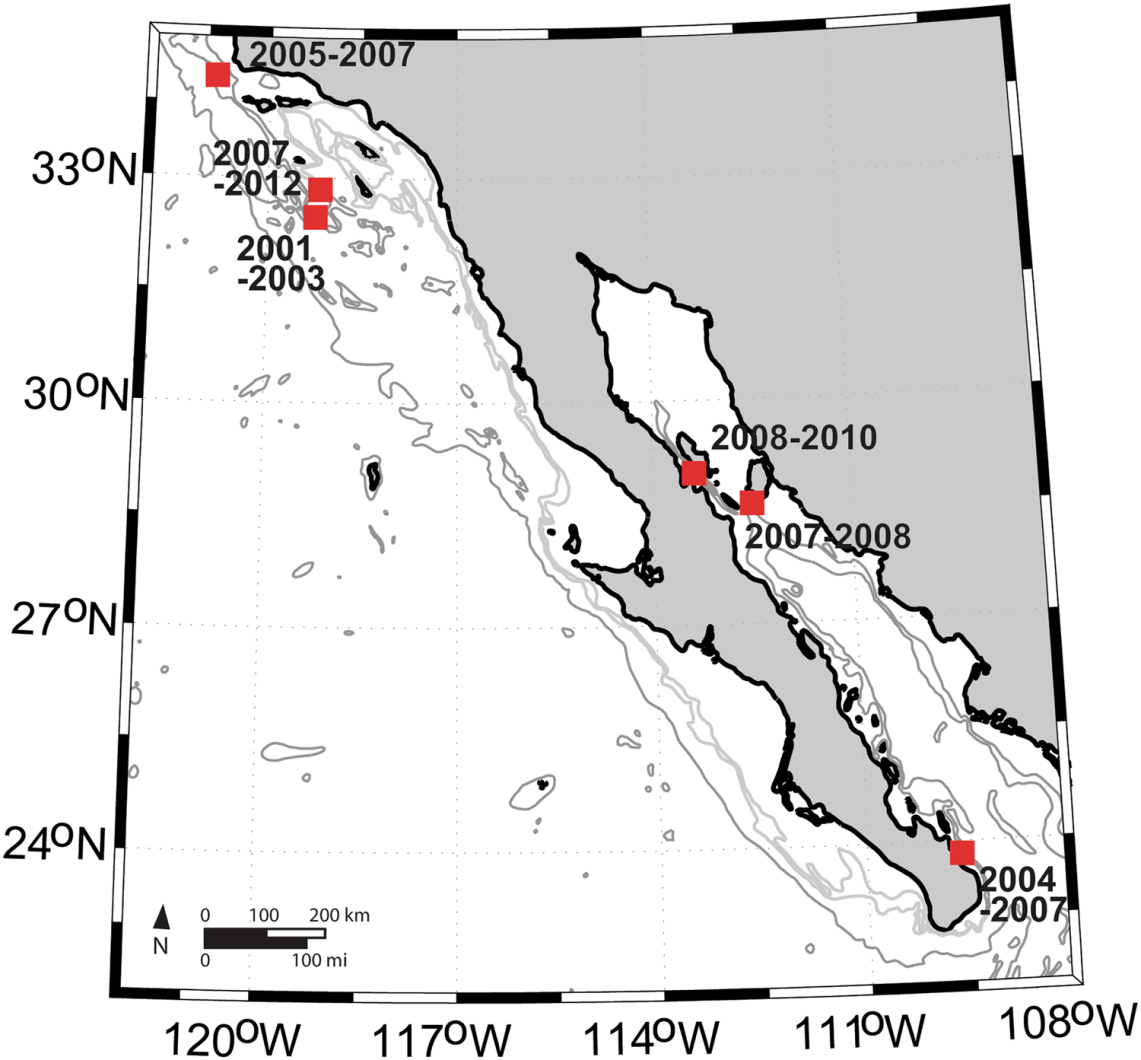
Recorder locations in Southern California and the Gulf of California, with years when data were collected at each site noted. Map generated using Matlab (https://www.mathworks.com/).

We used multi-year recordings from Southern California and the Gulf of California (Fig. 1) to describe different fin whale song types present in these areas based on their IPIs. We tested whether their seasonal (monthly) and long-term change patterns can be described using generalized additive modeling (GAM) framework. Based on these patterns, we postulated hypotheses about residency and movement of fin whale populations in these regions.

## Results

**Figure 2:**
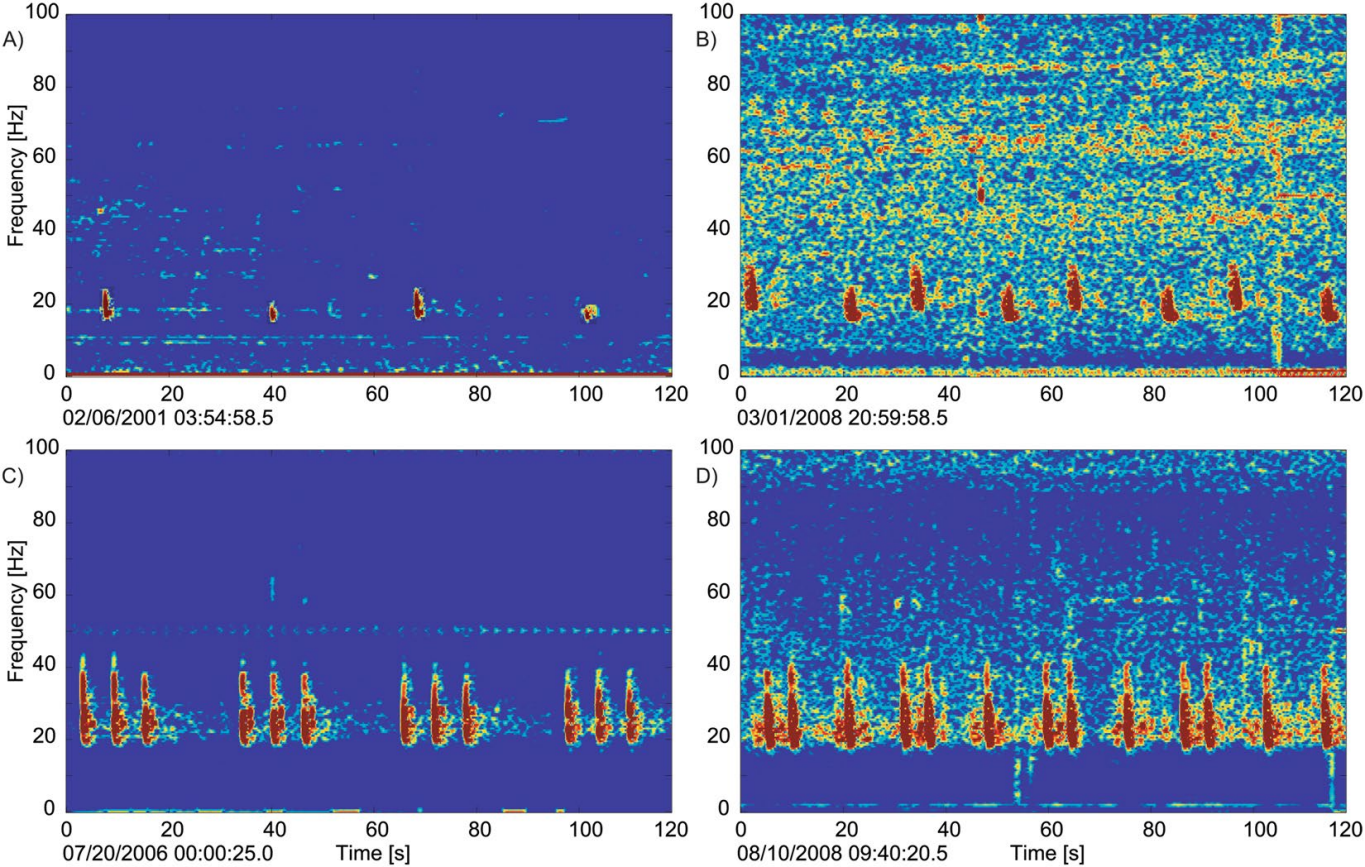
Examples of fin whale songs recorded in Southern California and Gulf of California. (**A**) long doublet; (**B**) short doublet; (**C**) long triplet; and (**D**) short triplet. All spectrograms were calculated using 4000-point FFT, 90% overlap, and Hanning window. Original data from which spectrograms were created is provided in Supplementary Information.

Four distinct song types were identified in the data and were termed long doublet, short doublet, long triplet, and short triplet based on the total IPI durations of the song and the patterns of intervals within the series. All four song types were recorded in the Gulf of California and three were heard in Southern California, though only two were common in each region: short and long triplets in the Gulf of California and short and long doublets in Southern California (Fig. 2).

### Song type characteristics and occurrence

**Figure 3:**
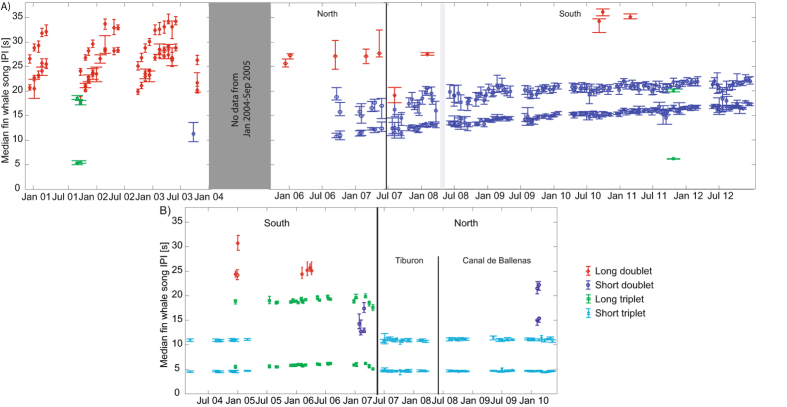
Long term trends in interpulse intervals (IPIs) of songs recorded in (**A**) Southern California and (**B**) Gulf of California. In Southern California, data were collected at two different locations, one more southerly (2000–2003 and 2007–2012) and one northerly (2005–2007). In the Gulf of California, data came from three different locations, one southerly (2004–2007) and two northerly (2007–2010). Only one area was sampled at any one time. The median intervals with 1^st^ and 3^rd^ quartiles (whiskers) are shown for each day with measured songs. Short doublets are marked as dark blue circles, long doublets are red diamonds, short triplets are cyan exes, and long triplets are green squares. Grey shaded area marks prolonged period without effort in the region and black lines delineate recordings from different sites.

The long doublet was previously described by Oleson and colleagues^10^ as having seasonally variable IPIs. This monthly pattern of longer IPIs during the winter months and shorter IPIs in the summer months was also shown to be the dominant descriptor within the GAM framework (Table 1). This song type was most common in Southern California during 2000–2003, though it was also occasionally heard in the southern Gulf of California before 2006 and more rarely in Southern California from 2005–2011 (Fig. 3A).

**Table 1:** Results of generalized additive models for short and long IPI of each song type that could be modeled.

	Long doublet		Short doublet		Short triplet	
	Short IPI	Long IPI	Short IPI	Long IPI	Short IPI	Long IPI
Month	93.38** (<*2e-16*)	100.6** (<*2e-*16)	N/A	N/A	N/A	N/A
Year	N/A	N/A	1044** (*<2e*-*16*)	177.1** (<*2e-16*)	N/A	*1*.966* (0.011)
N	23	23	116	116	42	42
R-squared	0.895	0.902	0.95	0.77	N/A	0.129
GCV	0.058	0.037	0.009	0.035	N/A	0.003

F-value and (p-value) are shown for significant variables with those that were significant at α < 0.05 marked with * and those at α < 0.001 marked with **.

**Figure 4:**
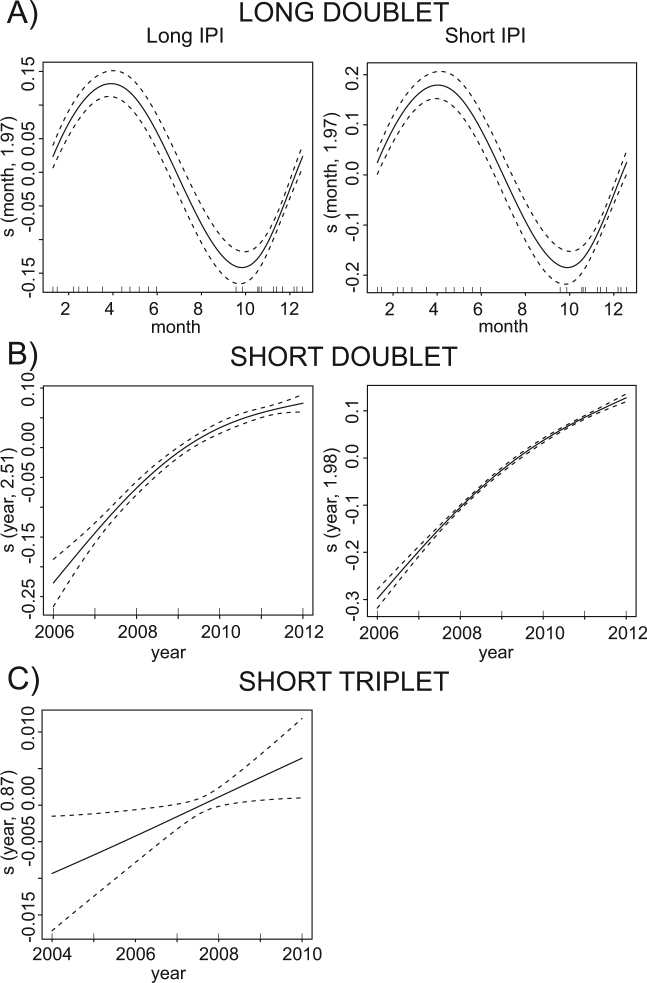
The mean-adjusted partial fit (solid line) and standard error fit (dashed line) of each significant explanatory variable for the generalized additive models describing temporal trends of different fin whale song types. The vertical lines along the x-axis indicate an observation at that value of descriptor variable. On the y-axis, s represents fitting via a spline function with name of variable and final number of degrees of freedom for the chosen model fit in parentheses.

The short doublet was the dominant song type in Southern California from 2006 onward (Fig. 3A). Its IPIs were substantially shorter than those in the long doublet, however, the short doublet IPIs were consistently lengthening over our monitoring period (Figs 3A and 4). These doublets were only occasionally recorded in the Gulf of California, and were more common at the southern than at the northern Gulf site.

The long triplet consisted of two shorter IPIs, identical in length (~6 s), followed by a longer one (~20 s; Fig. 3A). This was the dominant triplet at the southern Gulf of California site from spring 2005 to spring 2007, when the recording at this location ended (Fig. 3B). It was the only triplet recorded in Southern California (twice in fall 2001 and once in winter 2011). Is it notable that the long triplet song initially exhibited a lengthening of the IPIs, similar to the long-term lengthening of the long doublet IPIs, though the IPIs decreased rapidly in winter 2007 (Fig. 3B). However, month and year were not significant variables to allow us to develop a model that describes the variability in the IPIs (Table 1), likely due to the small sample size and few years of data when this song type was recorded in our data in the Gulf of California.

The short triplet song type consisted of one short (~5 s) IPI and two longer IPIs of similar length (~10–11 s). The short triplet was the dominant call from winter 2004 to spring 2005 at the southern Gulf of California site, and it was the only triplet recorded at the northern Gulf of California sites (Fig. 3B). The IPIs measured from the short triplet did not vary monthly, but both short and long IPIs changed over the years (Table 1). The short triplet long IPI consistently increased, while the short IPI duration peaked in 2007 but then started decreasing (Fig. 4). The short triplet was not heard in Southern California. With the exception of only one day in December 2004, there was no overlap between short and long triplet songs in the Gulf of California.

There was a qualitative difference in characteristics of the pulses that comprise songs in these two regions. Southern California doublet songs were comprised of two alternating pulses (notes and backbeats) with different frequency characteristics (Fig. 2). The bandwidth and frequency characteristics of the 20 Hz pulses forming Gulf of California songs were consistent across the triplet song (Fig. 2).

### Sequence variability

Among the four major song types there occasionally was substantial variability in the sequencing of the IPIs. Doublet songs also included singlets made up of one of the doublet IPIs. Of the days with short doublet song occurrence, 18% had singlets, while 71% of days with long doublet song had singlets. In long doublet song sequences, the longer IPI of the series was generally repeated as a singlet, whereas in the short doublet, the shorter IPI was more commonly repeated as a singlet. Notably, the long singlet (singlet variant of the long doublet) was more common than the long doublet at both the southern Gulf of California site and the later recording period in Southern California (Fig. 3). Triplet songs could also vary in their IPI sequencing, with nearly all analyzed days consisting of doublet sequences with the same IPIs as triplets, interspersed with the triplet song. Additionally, about 25% of days with triplet song also featured quadruplets, pentuplets, or more repeated IPIs. Triplet song variants always featured multiple repeats of the most dominant unit with only the number of repetitions varying, often within an individual song sequence.

### Spatial and temporal patterns in song types

**Figure 5:**
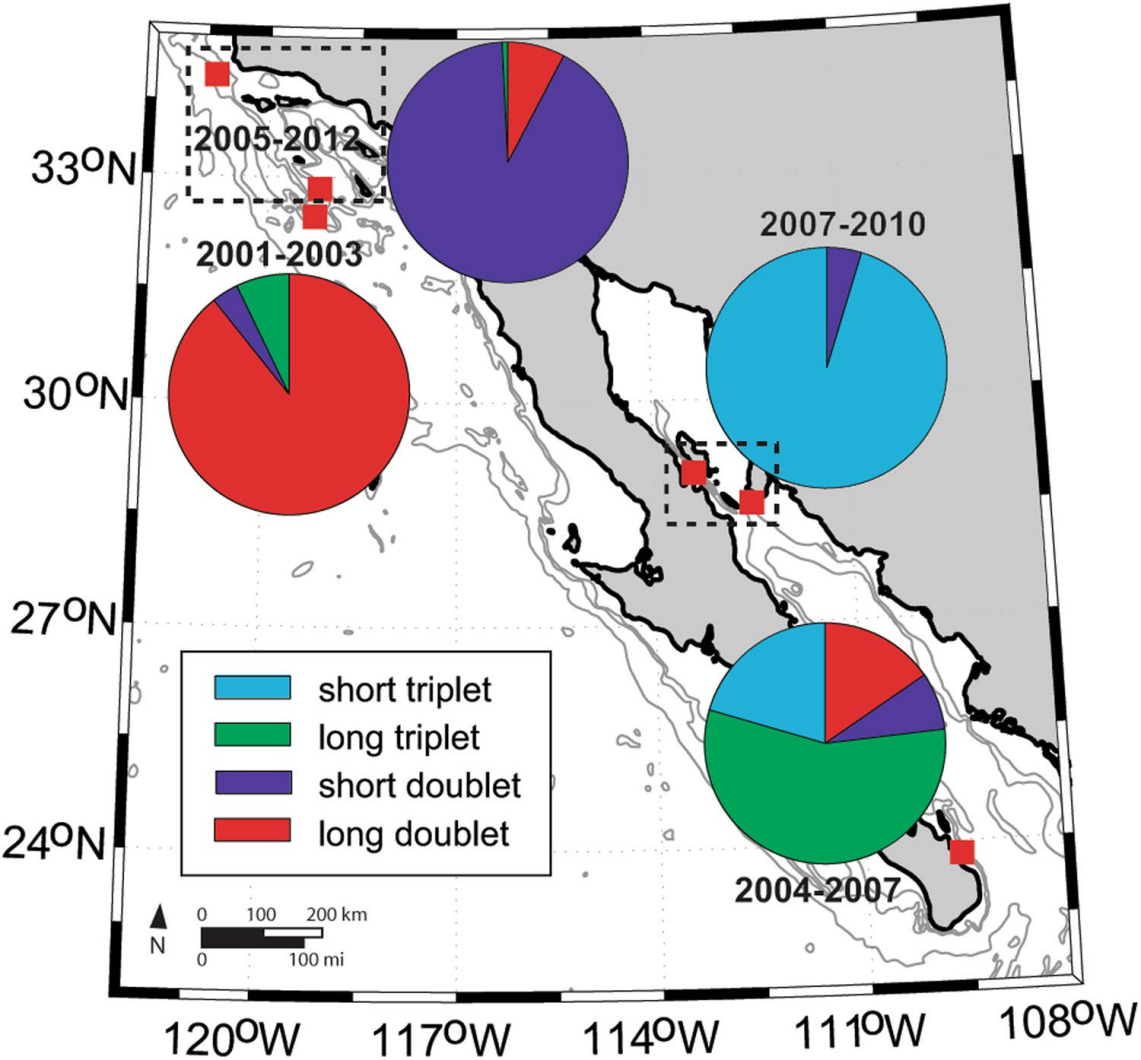
Spatial variability in song types in distinct geographic regions and time periods. In Southern California, data from two sites with data from 2005–2012 were grouped and in Gulf of California northern sites with data from 2007–2010 were grouped (groupings denoted with a box). Map generated using Matlab (https://www.mathworks.com/).

**Figure 6:**
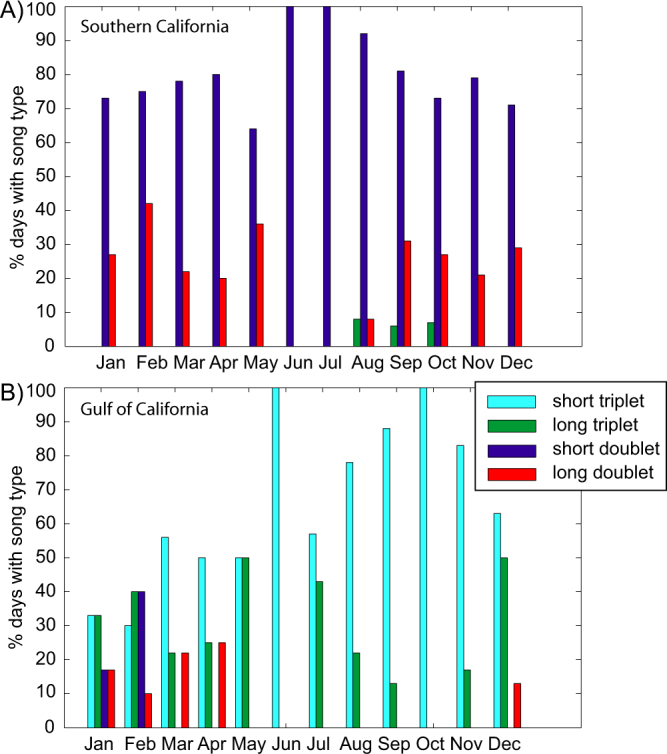
Percent of days with analysis effort for each month that contained a particular song type in (**A**) Southern California and (**B**) Gulf of California.

There was some spatial separation and difference in occurrence among the four song types. For example, the long doublet and long triplet songs were never recorded in the northern Gulf of California, but occurred at the other locations (Fig. 5). The short doublet, on the other hand, was recorded in all areas. The short triplet had the most restricted range, occurring only in the Gulf of California (Fig. 5). In addition to spatial variability, there were distinct seasonal patterns to the different song types in these two regions (Fig. 6). The long doublet was present during most of the year in Southern California with a peak in the winter, but it was absent in June and July. The short doublet was present year-round with peak occurrence in June and July. The long triplet was detected occasionally in Southern California from August to October. Both triplet songs were detected in the Gulf of California year-round with a summer-fall peak for the short triplet, and bimodal winter and spring/summer peaks for the long triplet. Long and short doublets were detected in the Gulf of California in the winter and early spring (Fig. 6).

## Discussion

Four distinct fin whale song types were present in Southern California and the Gulf of California between 2000 and 2012. Even though multiple song types were detected in both regions, in any given area during any one time, only one song type was dominant. In Southern California, the dominant song was always a doublet and in the Gulf of California it was a triplet. Within each region there was an abrupt switch between predominant song types. In Southern California there was a relatively sudden change in the dominant song type from the long doublet to the short doublet in 2006. Similarly, in 2005 in the southern Gulf of California, the dominant song switched from the short triplet to the long triplet. Additionally, our data provide an example of a gradual, consistent, long-term and population-wide slowing down of song beat (or an increase in IPI). Below, we address possible reasons for the observed song type switch and long-term song modification and discuss whether these patterns may indicate a change in population that is most common in the monitored area or if they imply song switching by a single population. Our data do not provide a definitive answer, but we argue that population change is the more likely reason due to a number of factors, including differences in seasonal and inter-annual patterns of each song type.

Our analysis of long-term trends revealed that each song type had distinct seasonality and inter-annual trends in IPIs. When the song type switched in an area, the temporal variability of the new dominant song type did not match patterns of the previous song type. For example, long doublet song changed seasonally, and reset annually^10^. It was replaced by a short doublet song with weak seasonal variability and a strong inter-annual increase in IPIs. Additionally, the seasonal occurrence of the doublet song types was quite different, with the long doublet song generally absent in summer whereas the short doublet song was common in that season. These changes indicate that fin whales in Southern California did not just change the characteristics of their song, but they also altered when they sing and how the song changes over time. Similarly, triplet songs in the Gulf of California changed from the long triplet that was more common during the winter and spring to the short triplet that was more common during the fall. A switch between song types on a seasonal basis could suggest different functions of the song within the same population. Our data indicate a major shift from one song type to another at a single point over the 12 year datasets, such that a change in song function seems an implausible explanation. Rather, it is more likely that the observed shift from one song type to another represents a shift in which population was using the monitored area, and that the shift persisted over several years.

The link between song style and population identity is also supported by the geographic patterns in the detection of each song type. Each of the song types observed within our dataset was heard primarily in one region, but three of the four song types were heard less frequently in the other region, thus suggesting seasonal or other occasional movements of each group. The long triplet from the Gulf of California was recorded in Southern California, and both Southern California doublet songs were recorded in the Gulf of California. Those observations suggest that even when individuals are in an area where their song is not dominant, they do not adopt the new song but continue singing their own song. In oscine birds, songs are often learned during early development which ends in song crystallization^3^. If adults retain the ability to adapt their song after maturity, such as in semi-flightless passerine (*Philesturnus carunculatus*)^27^, they are engaging in open-ended vocal learning. Evidence for vocal learning in baleen whales is rare and is largely limited to changes in signal frequency^28^. One exception are humpback whales which synchronously change their songs across populations^29^, and include one documented occurrence of a population adopting the song of a new immigrant into the area^30^. Unlike the case of humpback whales where the whole population adopted the new song, we detected long doublet song for years after it was no longer the dominant song in Southern California. Several questions remain about the links between song and population identity in fin whales: how long are songs retained by an individual after moving into a new area; are excursions to new areas temporary or do individuals stay in new areas? To answer these questions, research matching an individual’s song characteristics to genetic or photo identifications over time is needed. Considering that occurrence of non-dominant songs in any region was a relatively rare event, however, this is likely to be a difficult and long-term task. In summary, along with the qualitative difference in characteristics of the pulses that comprise songs in these two regions, we argue all of the above factors point to a likely distinction in song types across populations.

If we accept the hypothesis that songs indicate different populations, we could infer that four fin whale populations occurred in Southern California and the Gulf of California. One population singing the long doublet song could be an indicator of a pan-Pacific fin whale population, since that song was also recorded concurrently off Hawaii and in the Bering Sea, as well as Southern California^10^. The short doublet could represent a Southern California resident fin whale population. There is recent evidence for an increase in the fin whale population in this area^31^, as well as its residence in the region^17^. The short triplet song described here is consistent with song previously recorded from a resident population in the Gulf of California^14, 16^. The long triplet is a new song and may indicate a population from a previously unmonitored area. The short doublet and short triplet song types are heard year-round in Southern California and the Gulf of California, respectively, and have similar seasonal peaks in occurrence (summer and fall). Such year-round presence of the dominant songs in each region supports the assertion that songs are produced by populations that are present year-round. Detection of these songs in other regions suggests some degree of movement among areas. The Gulf of California triplet song was heard in Southern California during the summer, while the doublet song from Southern California occurred in the Gulf of California during the winter and spring. In both cases, this pattern is consistent with the general baleen whale migration patterns: to higher latitudes during the summer and lower latitudes during the winter^32^. The occurrence of non-dominant songs, especially doublets in the Gulf of California, can persist in the non-dominant area for a couple of months. During that time, the non-dominant song did not show any change in pattern that would indicate adaptation to the dominant song type of the region. Observations of song types across a broader geographic region could help elucidate whether these song types truly represent populations that are primarily resident to a relatively smaller area.

The patterns of songs in these two regions may point to existence of fin whale populations with different ecological characteristics, with one group possibly occupying a more coastal niche and the other occurring more offshore. Mitogenomic analysis, while not conclusive, pointed to two possible lineages in the North Pacific fin whale subpopulation^18^. Satellite tracking of fin whales from Southern California indicates that some animals may be more likely to move coastally while others travel further offshore^33^. Acoustic work from other locations in the Northeastern Pacific also may show separation of coastal and offshore groups. For example, fin whale song recorded along the Juan de Fuca Ridge from 2003 to 2006 consisted of pulses repeated at 25 and 30 s IPIs^22^, consistent with the pan-Pacific long doublet song^10^. The same song was also recorded off British Columbia in 2010/2011, in addition to a different song, with IPIs ranging from 11–13.4 s and 15–17.7 s and gradually increasing over time^34^. That latter song is not identical to the Southern California song, as the IPIs are shorter in songs off British Columbia, but it is more similar to the Southern California short doublet than the pan-Pacific long doublet song. These different songs may point to a possible distinction in songs between coastal and offshore fin whale populations across a broad swath of the Northeastern Pacific Ocean.

On a finer scale, recent visual surveys in Southern California indicate seasonal movements of animals in this region. During the winter, fin whales are sighted inshore and during the spring and summer they appear to move offshore^35^. Examination of fin whale songs across inshore and offshore sites in Southern California might shed light on whether these seasonal movement patterns are reflected in song occurrence and whether they also indicate further population structure. Analysis of seven years of data (2006–2012) across Southern California showed generally higher presence of fin whale calls (not necessarily songs) in the southwestern offshore region and peak detections in the winter and spring^17^. Data analyzed for this paper were primarily recorded on what would be considered offshore locations and we detected songs year round. More detailed information on genetics and seasonal singing behavior across the Southern California region is needed to better understand regional population dynamics.

Even though we believe songs identify populations, modifications within some song types, either seasonally or long-term, may make it challenging to use song as a tool to monitor populations. Such changes within songs suggest it may be difficult to associate a song recorded during any short time period to a specific population unless there is a comprehensive catalog of song types and their characteristics. For example, one of the few papers that presented long-term time series of fin whale song patterns only included two years of data^20^. During that time period, there were two instances of a sudden change in IPIs in the spring, indicating this is likely a regular occurrence. Similarly, we noted a sudden decrease in IPIs in the long triplet song at the end of the recording at the southern site in the Gulf of California. Unfortunately we do not have additional data to evaluate whether this is a singular or regular occurrence in this area. Despite these challenges, it is clear that song types are reasonably consistent over long periods and may be reliable indicators of the occurrence of a population. Song with IPIs consistent with the long triplet is reported from recordings from the Gulf of California from 1987^14^. This song appears to have changed little over that period. Multi-year data will be needed from multiple locations across a region to fully understand fin whale song dynamics.

Assuming fin whales did not switch their song, but these sudden changes in 2005 in the Gulf of California and 2006 in Southern California indicate a shift in the occurrence of a population, why did the populations suddenly shift? We know from previous reports that abrupt disappearance of songs can occur. For example, in the Gulf of California, a fin whale song with 9–12 s IPIs was recorded in 1985, but it was not reported again in 1987^14^. One possible explanation is a change in environmental conditions. Fin whales are thought to be opportunistic feeders^36^, but if population-specific food preferences exist they could drive such population movements. Prey changes, driven by events such as the El Niño Southern Oscillation, are known to affect the distribution of other baleen whale species^37^. More detailed information on the food preferences of different fin whale populations would be needed to evaluate if changing ocean conditions, such as for example the occurrence of a brief La Niña in 2005/06^38, 39^, could have caused a shift in these populations.

Beyond using song as a tool for population identification, it may also be possible to identify individual whales by evaluating differences in song sequencing, or variations in amplitude and frequency between song sequences. It has been shown that small deviations in song pattern can indicate individual identity in birds^40^, so the same may be true for other animals. For fin whales, that could mean different patterns in song complexity such as variable arrangements of singlets, doublets, and triplets throughout a song. While we observed significant variability in sequencing within each song type, we did not perform additional analysis on these features because we could not make confident assumptions on the individual producing consecutive sequences. Individual variability may also be apparent as variation in amplitude or frequency of the signal. We did not analyze these features for this work because autonomous recorder data are not ideally suited for such detailed analysis as it is not possible to know how much variability in those features results from propagation effects versus actual individual variability. Acoustic tags deployed on calling whales would be a better place to start teasing apart these finer scale patterns and answering individual-level questions.

Some of the challenge in interpreting our data comes from changes of recording location over time, both in Southern California and the Gulf of California. For example, it would have been informative to have concurrent recordings in the southern and northern parts of the Gulf of California. Also, more recent recordings from those areas could indicate whether the triplets recorded during 2000s have persisted or if they have changed. In Southern California, the gap in recordings during 2004 happened during a time of change in song types. Analysis of a more northerly site in 2005 and 2006, since those were the only available data from the region during that time, may have introduced some small-scale regional complexity to our data interpretation. However, preliminary analysis of data from the more northerly site since 2006 shows patterns that very closely match those at the southern site reported in this paper, therefore we believe the sites to be comparable. In any case, consistent recordings from a single site over a long time scale would reduce uncertainty in interpretation that occurs when time and space get confounded due to gaps in recordings.

Another intriguing question coming from these data is how do fin whale populations synchronize song over large scales and gradually modify the IPI. This synchrony was also noted for fin whale songs across an ocean basin^10^. When rhythm synchronization was studied in other organisms, synchronization of faster rhythms (>1 Hz) was typically investigated. The ability to anticipate such rhythms has been observed only in humans and a few animal species, including a California sea lion (*Zalophus californianus*)^41^. However, the neural mechanisms involved in subsecond and suprasecond timing are quite distinct^42^. Even though perception of rhythm is not common among animals^43^, some examples of broad synchrony in rhythmic communicative signals include songs of whales^44^ and indris (*Indri indri*)^45^ or footdrumming of kangaroo rats (*Dipodomys spectabilis*)^46^. We are not aware of examples of rhythm synchronization over long time periods or of long-term systemic drift in the song rhythm. However, synchronization typically indicates a strong social interaction and may show group unity, bonding, and increased cooperation as generation of a complicated rhythmic display is perceived as an honest signal of coordination and time together^47^. In addition, matching of changing songs indicates a level of cultural transmission^48^. Cultural transmission has been documented in other baleen whales; for example, elaborate songs of humpback whales are modified through horizontal cultural transmission^29^. More detailed data on the interactions between singing fin whales would be necessary to shed light on mechanisms for this synchronization, which in turn may lead to a better understanding of the ecology, reproductive biology, and social structure of these populations.

Fin whale songs exhibit more complexity than evident from cursory investigations and our understanding of their occurrence and meaning is still rudimentary. In the future, to move beyond description of the songs themselves, studies that incorporate a larger variety of data (e.g. acoustic tags and visual observations), as well as approaches from neuroscience, behavioral studies, and ecology, would be most likely to lead to an increased understanding of this complex system, how it evolved, and its importance to the survival of this endangered species.

## Methods

Fin whale song patterns were analyzed from passive acoustic data collected using autonomous recorders in Southern California and the Gulf of California. Data collection in Southern California occurred at Tanner Bank from 2000–2003, off Point Conception from 2005–2007, and finally west of San Clemente Island from 2007–2012. In the Gulf of California, three different sites were used as well, a southern one from 2004–2007 and two northern sites from 2007–2010 (Fig. 1). Data from 2000–2003 were collected at 1,000 Hz sample rate using Acoustic Recording Packages^49^ and they were analyzed following procedures described in Oleson *et al*.^10^. Since 2004, all data were collected at higher sample rates using High-frequency Acoustic Recording Packages^50^. While most recordings were collected continuously at 200 kHz, several data sets were recorded on a duty cycle and at a lower sample rate (Table 2). All the data were downsampled to have an effective sample rate of 2,000 Hz, allowing consistent processing across the datasets.

**Table 2:** Dates, times, and locations of different recordings with data sampling protocol (sample rate and duty cycle shown as cycle interval-recording duration in minutes) indicated for each deployment.

Region		Latitude and longitude (°)	Recording start time	Recording end time	Sample rate (kHz)	Duty cycle (min)
Southern California	South	32° 41.3′ N 119° 01.9′ W	August 2000	November 2003	1	Continuous
	North	34° 18.9′ N 120° 48.1′ W	October 2005	June 2007	200	15-5 first two months, then continuous
	South	32° 50.8′ N 119° 10.6′ W	July 2007	September 2007	200	Continuous
		32° 39.4′ N 119° 28.4′ W	October 2007	March 2008	200	20-5
		32° 50.8′ N 119° 10.6′ W	June 2008	December 2012	200	Continuous
Gulf of California	South	23° 49.8′ N 109° 37.8′ W	March 2004	November 2004	200	40-5
			November 2004	April 2005	80	20-10
			May 2005	September 2005	200	25-5
		23° 55.6′ N 109° 40.8′ W	November 2005	January 2006	200	Continuous
		23° 49.7′ N 109° 37.8′ W	February 2006	June 2007	200	25-5
	Tiburon	28° 36.4′ N 112° 30.3′ W	June 2007	March 2008	200	20-5
	Canal de Ballenas	29° 1.6′ N 113° 22.5′ W	August 2008	December 2008	200	20-5
			April 2009	May 2010	200	15-5

To determine fin whale song patterns throughout our data, we randomly chose two days per month with fin whale songs; one day before the 15^th^ of the month and one after the 15^th^. For the given day, the start time of each 20 Hz pulse in a sequence was picked from a spectrogram and saved to a spreadsheet. Pulse start times were picked only when a clear IPI pattern was visible for a period of a minimum of 2 min. If two whale songs overlapped but were clearly distinguishable due to differences in intensity or spectral features, only one was picked at a time. When too many whales were calling, to allow clear distinction of an individual song, 20 Hz pulse start times were not picked from that time period. If no fin whale song that could be picked was identified during the selected day, we used a nearby day with identifiable song. Spectrogram display parameters were set to 60 or 120 s of data, and frequency range 0–150 Hz, with 1 Hz frequency and 0.1 s temporal resolutions.

After all the 20 Hz pulse start times were picked in a day, interpulse intervals (IPIs) were calculated for each picked sequence. Sequences with similar IPI patterns during each day were identified during this post-processing. First, the arrangement of pulses occurring together with similar timing (i.e. singlet, doublet, or triplet) was assessed to create song categories. Then IPIs of songs in the same category were pooled and sorted to separate short and long IPIs, which typically did not overlap. The daily median, and first and third quartiles for each IPI of each distinct sequence type were calculated. Based on the medians, songs were further classified into short or long versions of the song.

Trends in IPIs for each song type were evaluated to examine how song patterns change across seasons and years. To describe this change, we developed generalized additive models (GAMs) of short and long IPIs of each song type using the mgcv library in RStudio statistical software package (Version 0.98.507). GAMs are extensions of generalized linear models; they allow the additive predictors to be described by nonparametric smooth functions^51^. The link function of the GAM relates the mean of the response variable to the predictor variable. Two predictor variables used for the descriptive models were month and year during which the IPIs were recorded. Since the median IPIs were not normally distributed, we assumed a quasi-Poisson distribution in the response variable (IPI) and a logarithmic link function. The year was fit as a spline with an upper limit of three degrees of freedom and month as a cyclical cubic regression with a limit of four degrees of freedom. Model results for each individual variable and both of them combined were evaluated and the best model fit was determined based on the generalized cross validation (GCV) score, while also minimizing the number of variables used. Only data from the dominant geographic region where the song was detected were used for this analysis.

In addition to temporal trends in IPIs, we investigated overall spatial and temporal patterns in song occurrence. We calculated the frequency of occurrence of each song type from pre- and post-2004 recordings in Southern California, and from southern and northern recording locations in the Gulf of California. The frequency was calculated as the number of days of occurrence of a particular song divided by the total number of days analyzed for that period and location. To evaluate seasonal patterns in song occurrence in both regions, we summarized the monthly occurrence of each song type at each location by calculating the percentage of days analyzed during which that song type was identified.

## Electronic supplementary material

Supplementary information
